# Data concerning the chromatographic isolation of bovine IgG from milk- and colostral whey

**DOI:** 10.1016/j.dib.2018.09.115

**Published:** 2018-10-05

**Authors:** Hans-Jürgen Heidebrecht, Bernadette Kainz, Roland Schopf, Klaus Godl, Züleyha Karcier, Ulrich Kulozik, Beatrix Förster

**Affiliations:** a Chair of Food and Bioprocess Engineering, Technical University of Munich, Germany; b ZIEL Institute for Food & Health, Technical University of Munich, Germany; c Molecular Animal Breeding and Biotechnology, Gene Center, Ludwig-Maximilian-University, Munich, Germany; d Bijvoet Center for Biomolecular Research, Utrecht University, Utrecht, the Netherlands

## Abstract

Data included are related to the research article “Isolation of biofunctional bovine immunoglobulin G from milk- and colostral whey with mixed-mode chromatography at lab and pilot scale” (Heidebrecht et al., 2018) [1]. Data show individual bovine whey proteins in flow-through and elution fractions using different chromatographic resins as well as different binding and elution conditions. The relevant analytical methods for individual protein detection were SDS-PAGE and reversed phase- high performance liquid chromatography. The focus of the data is on the two mixed mode materials MEP HyperCel^™^ and Capto^™^-multimodal chromatography. Resins were used individually, in series and at different scale. Data provide information at which binding and elution conditions it is possible to isolate bovine IgG from milk and colostral whey and at which purity.

## Specifications table

**Table:** 

Subject area	*Chemistry, biology*
More specific subject area	*Isolation of bovine antibodies*
Type of data	*Graphs, figure*
How data was acquired	ÄKTApurifier 100 UPC, ÄKTApilot, Bio-Rad process chromatography station, *Zetasizer Nano ZS, SDS-PAGE, reversed phase-high performance liquid chromatography*
Data format	*Analyzed*
Experimental factors	*Fat (centrifugation), casein (microfiltration), lactose/minerals (ultrafiltration) removed from raw colostrum or milk to obtain whey*
Experimental features	*Determination of individual whey proteins in flow-through and elution fraction using different binding and elution conditions*
Data source location	Technical University of Munich *(Freising) and Ludwig-Maximilian-University (Munich) Germany*
Data accessibility	*With this article*
Related research article	*Data is provided as additional material directly related to the article*[1].

## Value of the data

• Isolation of bovine IgG from milk and colostral whey using the two mixed mode materials MEP HyperCel^™^ and Capto^™^ at different scale.
• Data deliver information about adsorption and desorption of bovine IgG at various different binding and elution conditions (pH, ionic strengths, buffer).
• Data are suitable for the setup of a chromatographic isolation process to obtain therapeutic amounts of isolated bovine IgG.

## 1 Data

The data show how to isolate bovine IgG from milk or colostral whey with two mixed mode materials and which conditions to use at different scale.

**Fig. 1:**
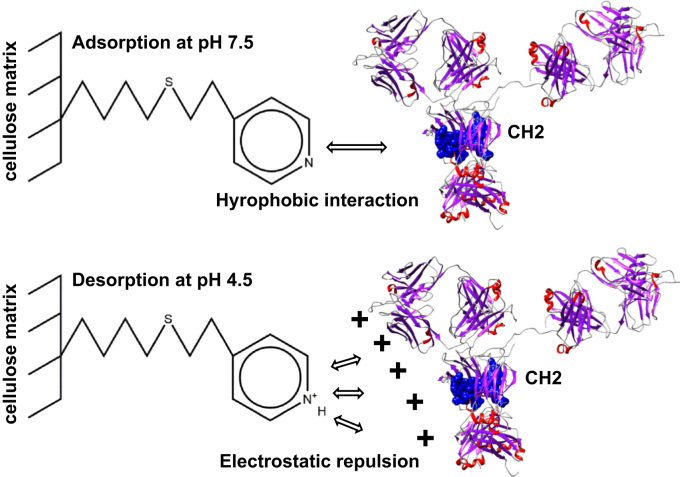
Adsorption and desorption mechanism of the MEP HyperCel^™^ column with the ligand 4-Mercapto-Ethyl-Pyridine (4-MEP) attached to a cellulose matrix. Adapted from [2]. The hydrophobic character of a protein is amongst others dependent on its secondary structure. Helical structures (red) are hydrophilic whereas β-sheets (purple) are comparatively hydrophobic. The light chain of IgG consists of 3% helical and 47% β-sheets and the heavy chain of 8% helical and 44% β-sheet structures, which is, amongst others, an indication of the hydrophobic character of IgG. Structural formula drawn with ChemDraw^®^. Structure generated with UCSF Chimera (pdb code 1HZH).

**Fig. 2:**
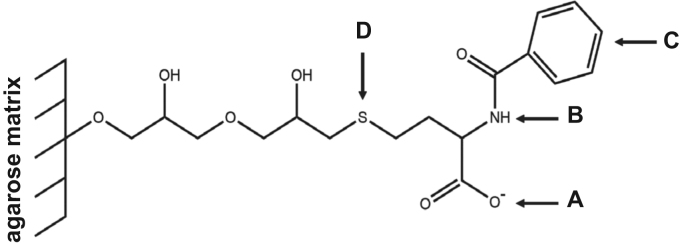
The ligand (N-benzoyl-homocysteine) of CaptoTM MMC with its multimodal functionality. The ligand offers interactions with a molecule based ionic interactions (A), hydrogen bonding (B), hydrophobic interactions (C), and thiophilic interactions (D). Structural formula drawn with ChemDraw^®^. Adapted from [3].

**Fig. 3:**
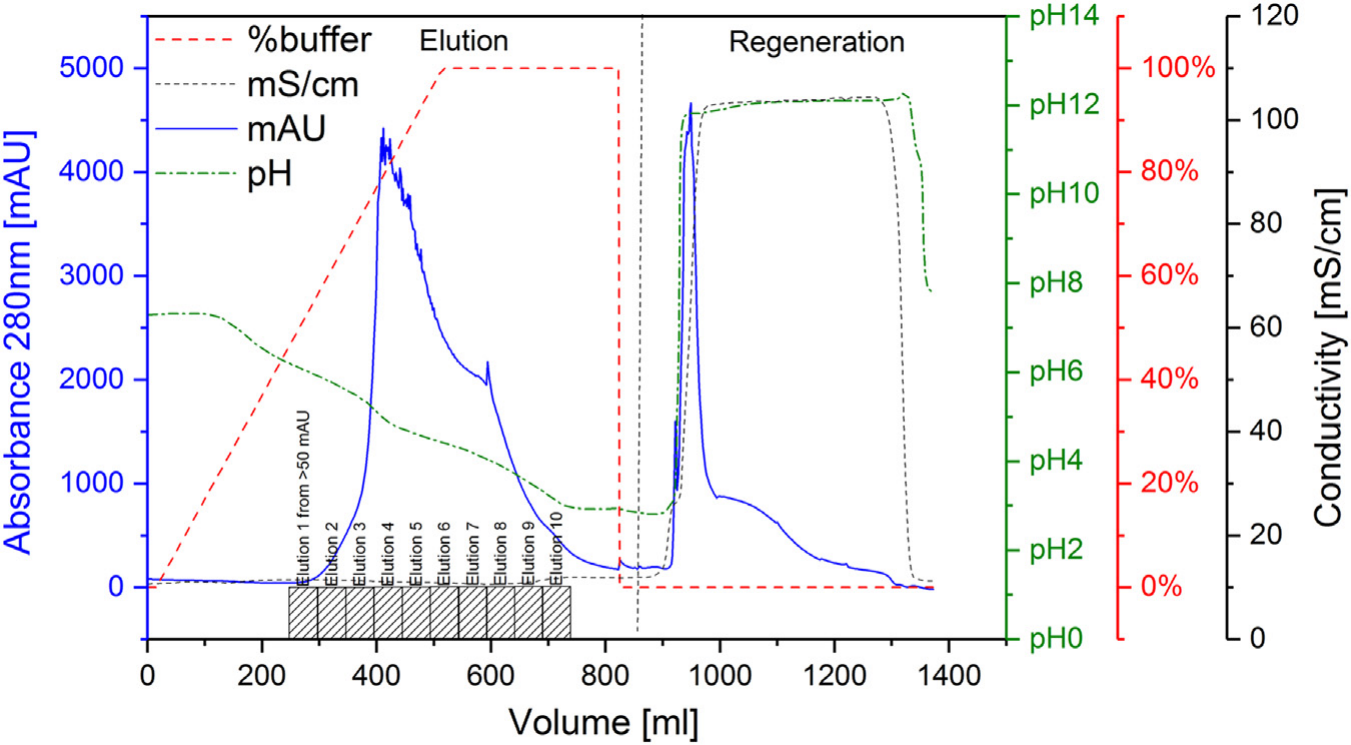
pH gradient elution using MEP HyperCel^™^ as column (100 mL) and whey obtained from milk as sample.

**Fig. 4:**
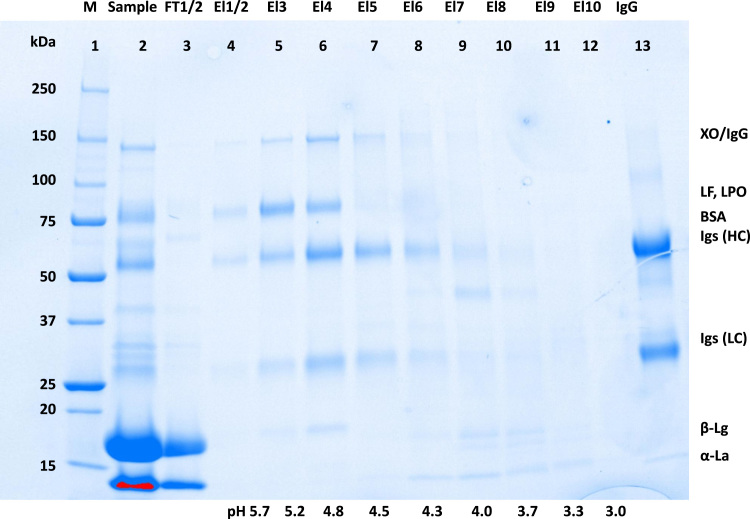
Reducing stained free SDS-PAGE (4–20 % gradient) of pH gradient elution shown in Fig. 3. Protein visualization with UV irradiation. Elution fractions equal to Fig. 3, lane 1 marker, lane 2 sample, lane 3 MEP-flow-through, lane 4–12 MEP-elution at different pH applied at four times concentration, lane 13 IgG standard.

**Fig. 5:**
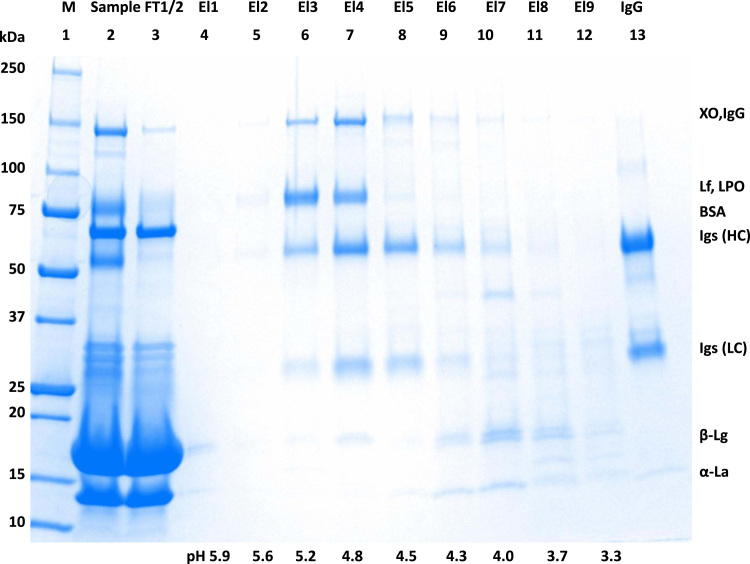
Reducing stained free SDS-PAGE (4–20% gradient) of pH gradient elution shown in Fig. 3. Protein visualization with coomassie. Elution fractions equal to Fig. 3, lane 1 marker, lane 2 sample, lane 3 MEP-flow-through, lane 4–12 MEP-elution at different pH applied at four times concentration, lane 13 IgG standard.

**Fig. 6:**
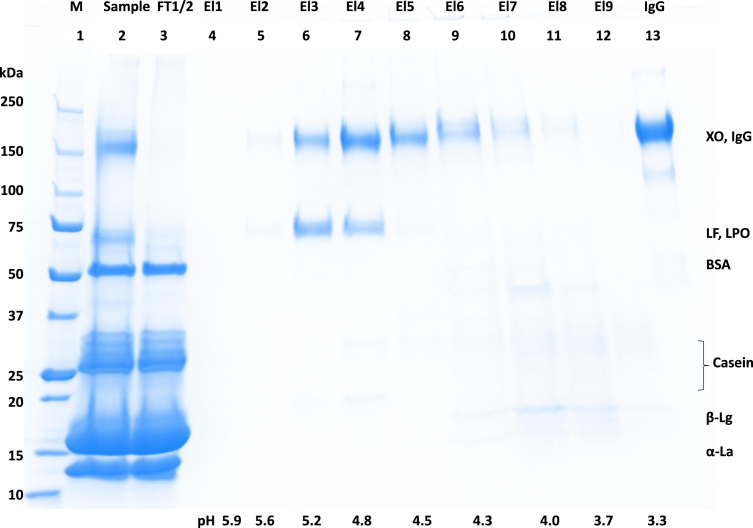
Non-reducing stained free SDS-PAGE (4–20 % gradient) of pH gradient elution shown in Fig. 3. Protein visualization with coomassie. Elution fractions equal to Fig. 3, lane 1 marker, lane 2 sample, lane 3 MEP-flow-through, lane 4–12 MEP-elution at different pH applied at four times concentration, lane 13 IgG standard.

**Fig. 7:**
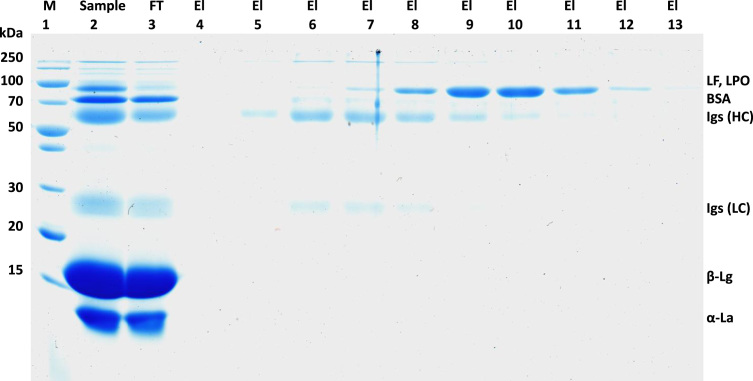
Reducing SDS-PAGE (15%) at increased pH (9 instead of 7.5) during binding (0.05 mol L^−1^ Glycin/NaoH pH 9) using Capto MMC column. lane 1 marker, lane 2 milk whey, lane 3 Capto MMC-flow-through, lane 4–13 elution at increasing ionic strength 0–100 % 0.05 mol L^−1^ Glycin/NaoH pH 9, 2 mol L^−1^ NaCl.

**Fig. 8:**
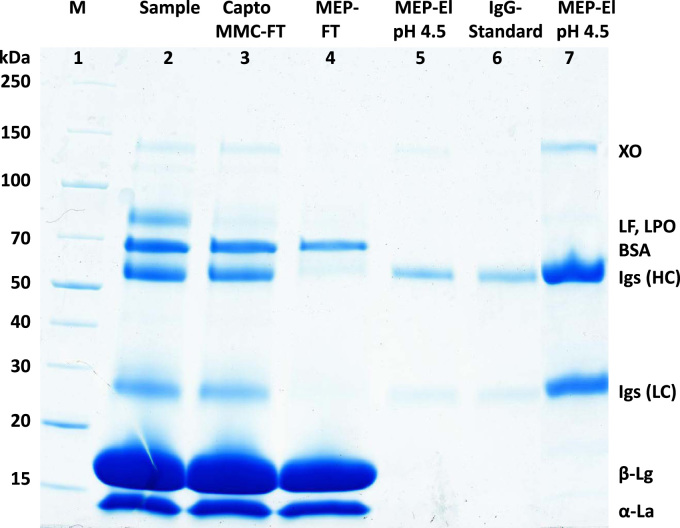
Reducing SDS-PAGE (4–15 % gradient) during cascade application of the Capto MMC-flow-through to the MEP column at adapted binding conditions (0.02 mol L^−1^ sodium phosphate/ 0.25 mol L^−1^ NaCl) lane 1 marker, lane 2 milk whey, lane 3 Capto MMC-flow-through, lane 4 MEP-flow-through, lane 5 MEP-elution (0.05 mol L^−1^ sodium acetate pH 4.5), lane 6 IgG-standard, lane 7 MEP-elution at pH 4.5 at fivefold concentration.

**Fig. 9:**
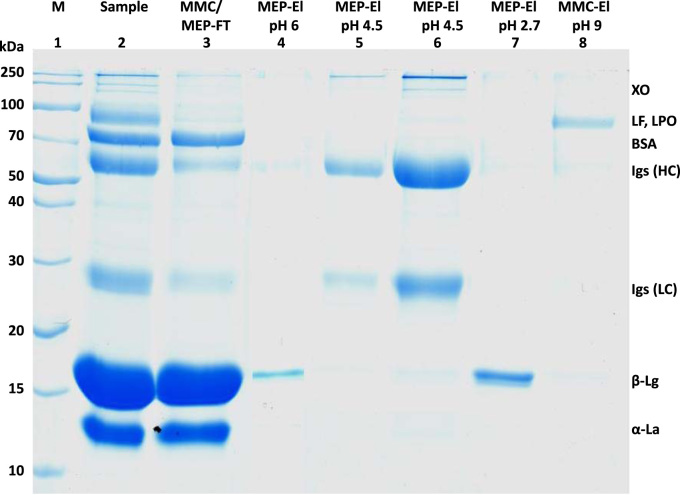
Reducing SDS-PAGE (15 %) during serial connection of the MMC and MEP resins and stepwise elution. Binding with 0.02 mol L^−1^ sodium phosphate/0.25 mol L^−1^ NaCl. Lane 1 marker, lane 2 milk whey, lane 3 MMC/MEP-flow-through, lane 4 MEP-elution at pH 6 (0.05 mol L^−1^ MES, pH 6) at twelvefold concentration, lane 5 MEP-elution at pH 4.5 (0.05 mol L^−^^1^ sodium acetate pH 4.5), lane 6 MEP-elution at pH 4.5 at sixfold concentration, lane 7 MEP-elution at pH 2.7 (0.1 mol L^-1^ Glycin/HCl pH 2.7) lane 8 MMC-elution at pH 9.0 (0.05 mol L^−1^ Glycin/NaoH pH 9, 2 mol L^−1^ NaCl).

**Fig. 10:**
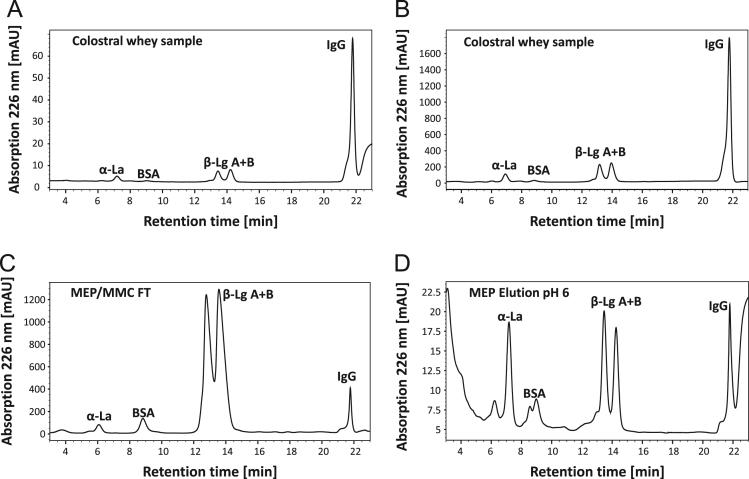
RP-HPLC chromatograms of the isolation process at highest scale MEP/MMC (8800 mL/300 mL): A: colostral whey for IgG detection, B: colostral whey for whey protein detection, C: MMC/MEP flow-through D: MEP Elution at pH 6; (because of different pre-dilution and injection volumes the comparison between the chromatograms is qualitative).

**Fig. 11:**
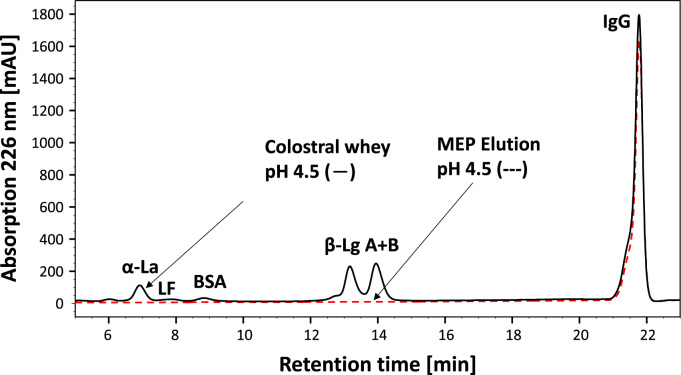
RP-HPLC chromatograms of the isolation process at highest scale MEP/MMC (8800 mL/300 mL), Colostral whey sample (solid), MEP primary elution fraction at pH 4.5 (doted).

**Fig. 12:**
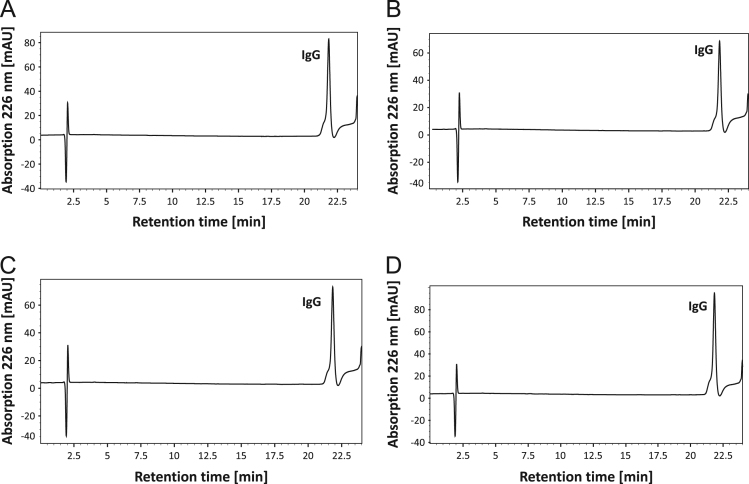
RP-HPLC chromatograms of the MEP primary elution fraction at pH 4.5 at highest scale MEP/MMC (8800 mL/300 mL) of four individual runs (A–D).

**Fig. 13:**
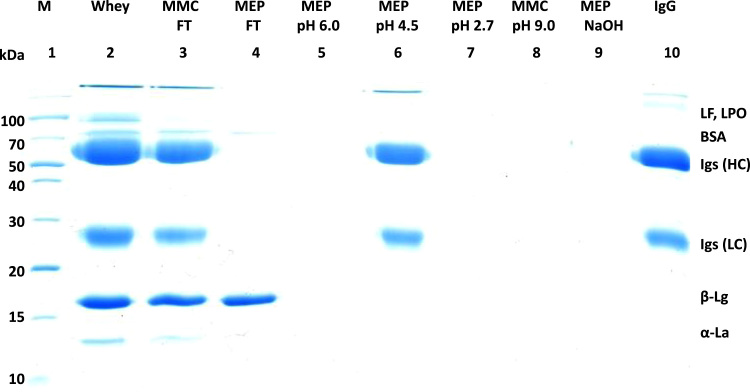
Reducing SDS of four individual runs at the highest scale MEP/MMC (8800 mL/3000 mL) (run A).

**Fig. 14:**
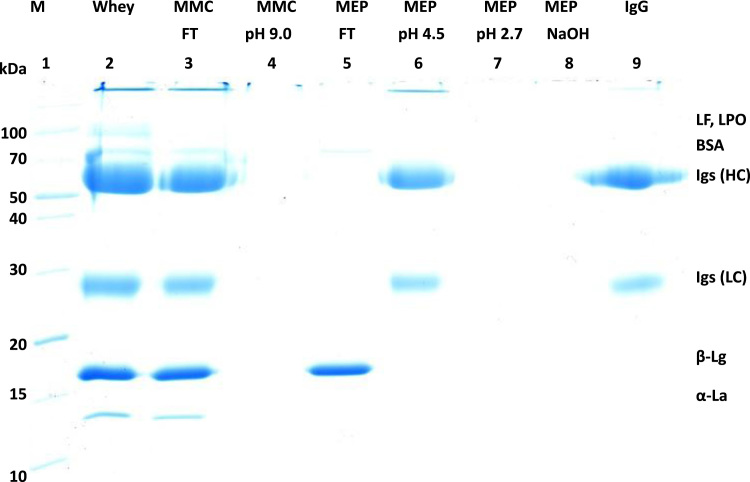
Reducing SDS of four individual runs at the highest scale MEP/MMC (8800 mL/3000 mL) (run B).

**Fig. 15:**
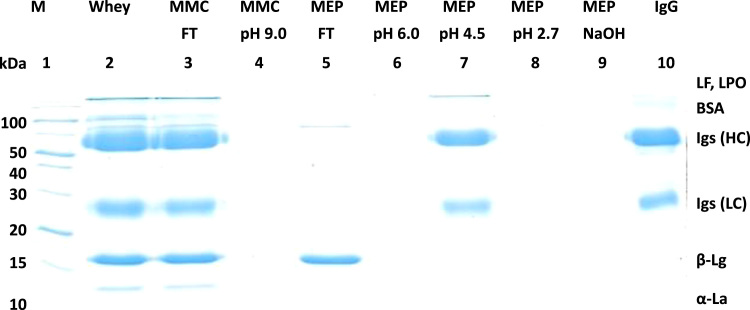
Reducing SDS of four individual runs at the highest scale MEP/MMC (8800 mL/3000 mL) (run C).

**Fig. 16:**
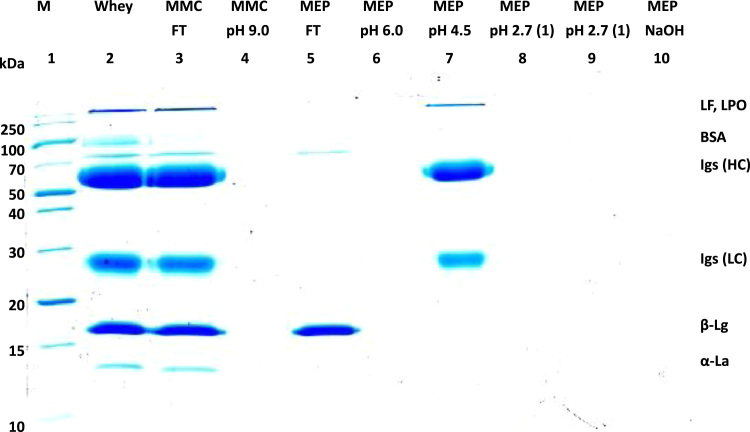
Reducing SDS of four individual runs at the highest scale MEP/MMC (8800 mL/3000 mL) (run D).

**Fig. 17:**
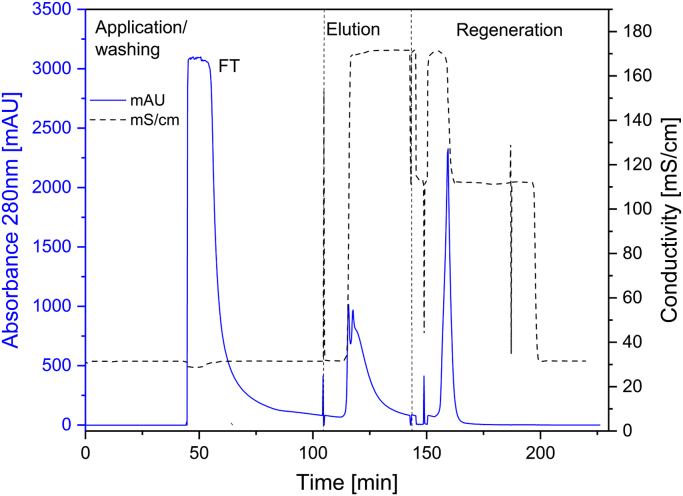
Progress of absorbance (solid) and conductivity (mS/cm, doted) as function of the time during sample application, elution and regeneration of a typical MMC run with colostral whey as sample at the highest scale (3000 mL).

**Fig. 18:**
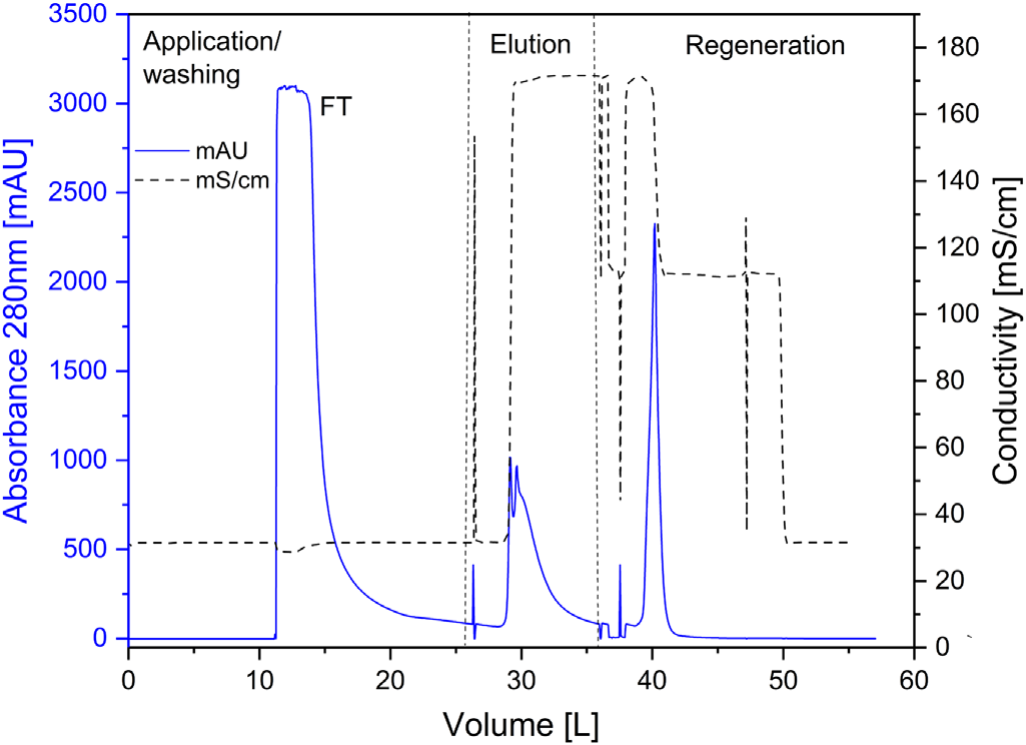
Progress of absorbance (solid) and conductivity (mS/cm, doted) as function of the volume during sample application, elution and regeneration of a typical MMC run with colostral whey as sample at the highest scale (3000 mL).

**Fig. 19:**
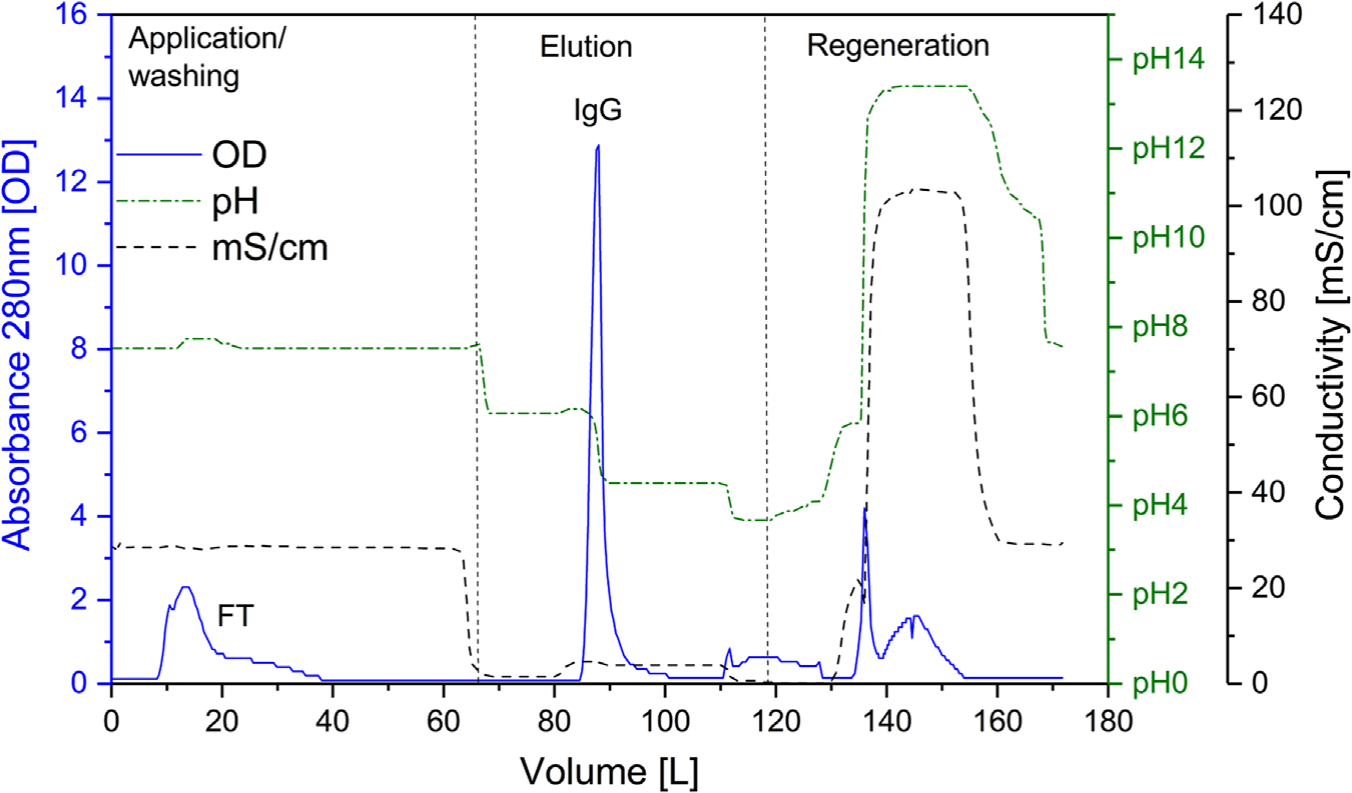
Progress of optical density (OD, solid), pH (solid/doted) and conductivity (mS/cm, doted) as function of the volume during sample application, elution and regeneration of a typical MEP run with colostral whey as sample at the highest scale (8800 mL).

**Fig. 20:**
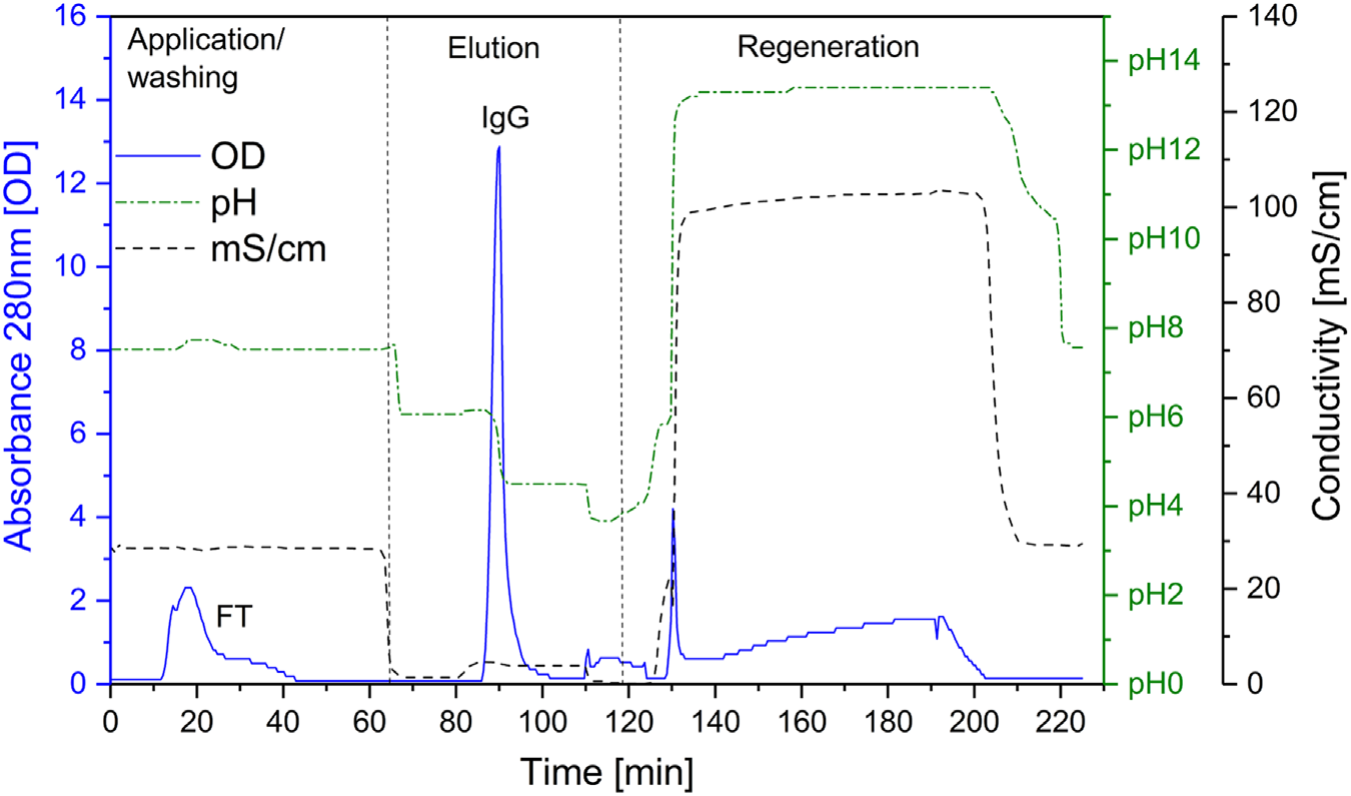
Progress of optical density (OD, solid), pH (solid/doted) and conductivity (mS/cm, doted) as function of the time, elution and regeneration of a typical MEP run with colostral whey as sample at the highest scale (8800 mL).

**Fig. 21:**
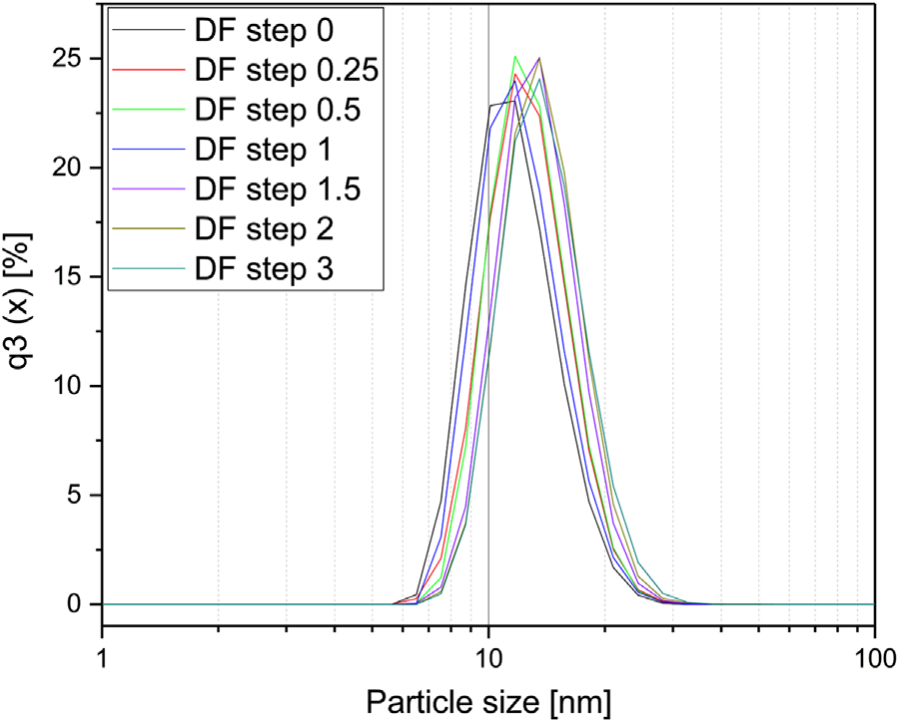
Particle size of IgG (MEP pH 4.5 elution fraction) at different diafiltration steps during desalting with distilled water.

Figs. 1 and 2 show the binding mechanism of the two resins MEP HyperCel^™^ (MEP) and Capto^™^-MMC (MMC). In order to determine the optimal elution pH of IgG from the MEP column a pH gradient elution was carried out (Fig. 3). To better detect the individual proteins the flow-through and elution samples, proteins were visualized with different methods on different SDS-PAGE gels (Fig. 4 reducing and non-reducing (Fig. 5) SDS-PAGE stained-free gel with UV protein visualization, Fig. 6 reducing SDS-gel with coomassie protein visualization). Fig. 7 shows binding and elution of IgG at increased pH (pH 9 instead of 7.5) during the binding phase at the MMC column. Fig. 8 shows the serial application of the flow through of the MMC column to the MEP column at increased ionic strength during the binding phase (0.25 mol L^−1^ NaCl). Fig. 9 shows the introduction of an elution step at pH 6 and a visible band that represents major whey protein β-lactoglobulin at the relevant lane. Fig. 10, Fig. 11, Fig. 12, Fig. 13, Fig. 14, Fig. 15, Fig. 16, Fig. 17, Fig. 18, Fig. 19, Fig. 20 show the process times and volumes, individual whey protein composition and purity of the different fractions of four individual runs of the developed isolation process at the highest tested scale MMC/MEP (3000 mL/8000 mL). Fig. 21 shows the change of particle size during the desalination the isolated IgG.

## 2 Experimental design, materials, and methods

The aim was to develop a novel and scalable process for the isolation of bovine IgG from colostral and milk whey, respectivley, with high purity and recovery. The preparation of the samples, the equipment and analytical methods for analysis are described in detail in [1].

For the isolation of IgG two resins were used. The resin MEP HyperCel^™^ (MEP) (Pall Corporation, Port Washington, USA) was used for direct capture of IgG, whereas the second material Capto^™^-MMC (MMC) (GE Healthcare, Uppsala, Sweden) was used for the removal of the minor whey protein lactoperoxidase (LPO). The data shown in Fig. 3, Fig. 4, Fig. 5, Fig. 6, Fig. 7, Fig. 8, Fig. 9 were carried out on an ÄKTApurifier 100 UPC (GE Healthcare, Uppsala, Sweden) with a tunable flow rate up to 100 mL min^−1^. The remaining isolation data in Fig. 10, Fig. 11, Fig. 12, Fig. 13, Fig. 14, Fig. 15, Fig. 16, Fig. 17, Fig. 18, Fig. 19, Fig. 20 were executed by an ÄKTApilot (GE Healthcare, Uppsala, Sweden) system with a flow rate range from 4 to 400 mL min^−1^ for the MMC resin respectively with an Bio-Rad process chromatography station (Bio-Rad Laboratories GmbH, Munich, Germany) with a flow rate range from 83 to 2000 mL min^−1^ for the MEP column. The corresponding buffers are summarized in Table 1.

**Table 1:** Overview of buffers used for the isolation of bovine IgG.

Buffer	Composition	Application	Manufacturer
PFA	0.03 mol L^−1^ disodium hydrogen phosphate	Gradient elution	Merck KGaA, Darmstadt, Germany
	0.03 mol L^−1^ sodium formate		Sigma Aldrich, St. Louis, USA
	0.06 mol L^−1^ sodium acetate		Merck KGaA, Darmstadt, Germany
NaOAc	0.02 mol L^−1^ sodium phosphate 0.25 mol L^−1^ NaCl	Binding buffer	Merck KGaA, Darmstadt, Germany
			Merck KGaA, Darmstadt, Germany
MES	0.5 mol L^−1^ 2-(N-morpholino) ethanesulfonic acid	Stepwise elution MEP	Merck KGaA, Darmstadt, Germany
pH 4.5	0.5 mol L^−1^ sodium acetate	Stepwise elution MEP	Merck KGaA, Darmstadt, Germany
pH 2.7	0.1 mol L^−1^ Glycin	Stepwise elution MEP	Carl Roth GmbH & Co. KG, Karlsruhe, Germany
	HCl		VWR International GmbH, Darmstadt, Germany
pH 9	0.5 mol L^−1^ Glycin	Elution MMC	Carl Roth GmbH & Co. KG, Karlsruhe, Germany
	NaOH		VWR International GmbH, Darmstadt, Germany
	2 mol L^−1^ NaCl		Merck KGaA, Darmstadt, Germany
NaOH	NaOH	Cleaning of columns	VWR International GmbH, Darmstadt, Germany

### 2.1 Particle size measurement during desalting

In order to monitor aggregation of IgG during desalting, the particle size was measured by dynamic light scattering using the Zetasizer Nano ZS (Malvern Instruments, Malvern, UK). At different diafiltration steps samples with constant IgG content were filtered using a syringe filter of 0.45 µm (Chromafil RC-45/25 Macherey-Nagel, Dueren, Germany). After a 5 min equilibration phase each sample was measured in duplicate at 20 °C. Each of the two runs consisted of 10 individual runs of 60 s (Fig. 21).
