# Supplementary material for: Data concerning the chromatographic isolation of bovine IgG from milk- and colostral whey
Source: Data Brief. 2018 Oct 5;21:527–39. doi: 10.1016/j.dib.2018.09.115 (PMC6199771; doi:10.1016/j.dib.2018.09.115)
Supplement: Supplementary file 1 — Supplementary material [file mmc1.docx]

**Conflict of interest**

We wish to confirm that there are no known conflicts of interest associated with this publication and there has been no significant financial support for this work that could have influenced its outcome.
